# Intraoperative challenges and management of fibrovascular membrane with tractional retinoschisis in proliferative diabetic retinopathy

**DOI:** 10.1186/s12886-024-03555-x

**Published:** 2024-07-20

**Authors:** Akihiko Shiraki, Nobuhiko Shiraki, Susumu Sakimoto, Kazuichi Maruyama, Takatoshi Maeno, Kohji Nishida

**Affiliations:** 1 Department of Ophthalmology, Osaka University Graduate School of Medicine, Suita, Japan; 2 Integrated Frontier Research for Medical Science Division, Institute for Open and Transdisciplinary Research Initiatives, Osaka University, Suita, Japan; 3 Department of Vision Informatics, Osaka University Graduate School of Medicine, Suita, Osaka Japan; 4 Premium Research Institute for Human Metaverse Medicine (WPI-PRIMe), Osaka University, Suita, Osaka Japan

**Keywords:** Retinoschisis, Fibrovascular membrane, Proliferative diabetic retinopathy, Intraoperative optical coherence tomography, Bimanual technique, Tractional retinal detachment

## Abstract

**Background:**

In severe Proliferative Diabetic Retinopathy (PDR), fibrovascular membrane (FVM) causes macular tractional retinal detachment (MTRD) which threatens vision and eventually leads to blindness. Here we present a case of separation between the inner and outer retina in tractional retinoschisis, induced during intraoperative FVM delamination.

**Case presentation:**

A 68-year-old woman presented with PDR in the right eye, characterized by a combined FVM and retinal detachment, for which a vitrectomy was performed. Multiple holes, large retinal detachment extending to all quadrants, and white-lined blood vessels with FVM were found during the procedure. When membrane delamination was performed, it strayed into the space between the inner and outer retinal layers without being noticed due to retinoschisis and multiple retinal holes. After removing the FVM and detaching the separated inner retina, fluid-gas and photocoagulation were performed. Retinal reattachment was successfully achieved after surgery, and the postoperative visual acuity was improved and maintained for 26 months postoperatively.

**Conclusions:**

When tractional retinoschisis due to FVM is combined with retinal holes in tractional retinal detachment (TRD), care must be taken to prevent delamination from straying into retinoschisis during separation.

**Supplementary Information:**

The online version contains supplementary material available at 10.1186/s12886-024-03555-x.

## Background

Proliferative diabetic retinopathy (PDR) is the main cause of vision loss and blindness in working age patients in developed countries [1, 2]. PDR is characterized by large non-perfused areas, and neovascularization causing various complications such as vitreous haemorrhage and fibrovascular membrane (FVM) [3]. In severe PDR, FVM causes macular tractional retinal detachment (MTRD) which threatens vision and eventually leads to blindness [4]. Therefore, when FVM complicates MTRD or tractional retinal detachment (TRD) located near the macula, vitrectomy is necessary to release the retinal traction. Particularly, when the space between the FVM and retina exists, delamination and bimanual techniques are often performed to avoid damaging the retina [5].

Here, we report a case wherein intraoperative separation was performed between the inner and outer retina in tractional retinoschisis during delamination.

## Case presentation

**Fig. 1:**
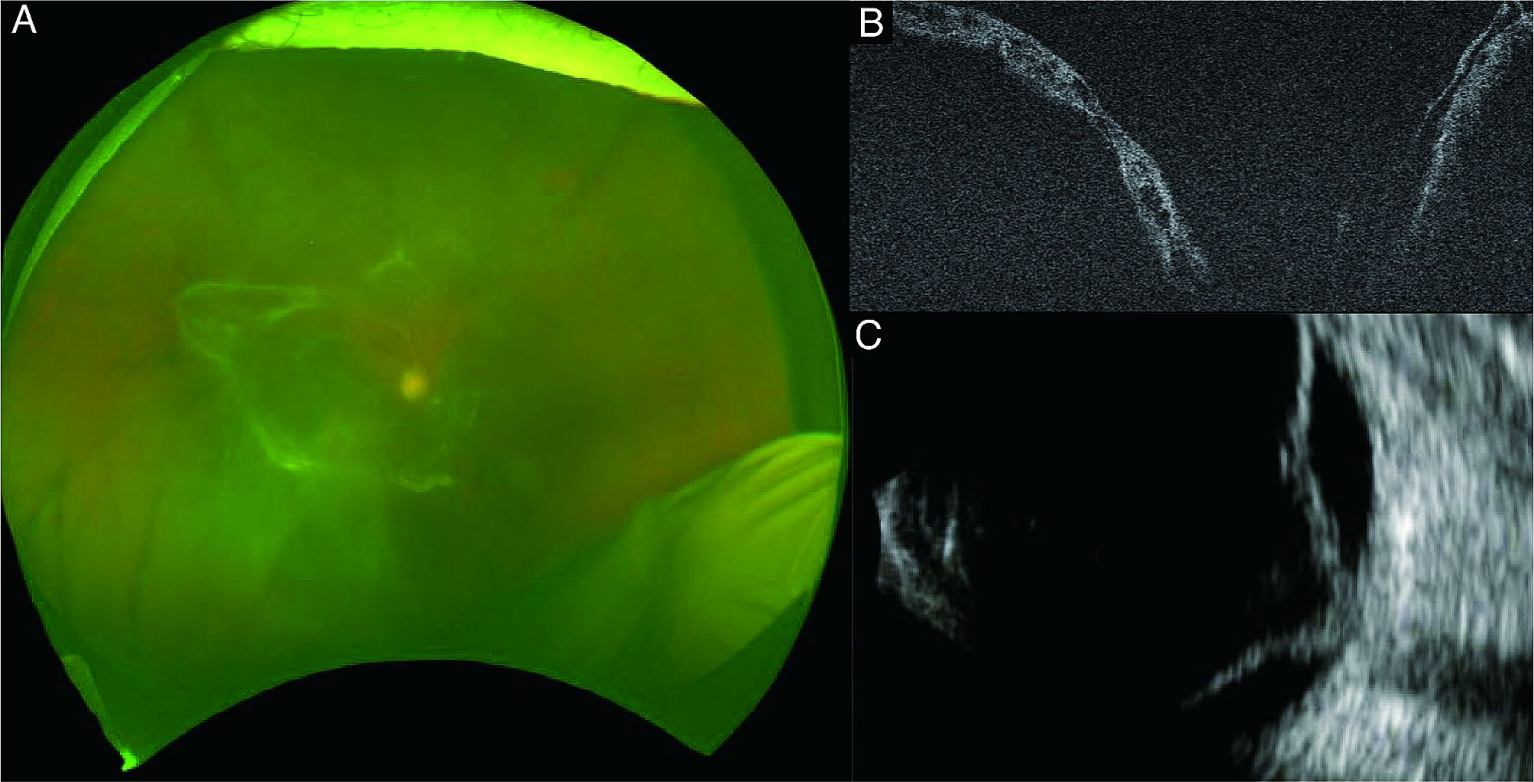
Preoperative images. **(A)** Retinal detachment and fibrovascular membrane (FVM) were found partially, but it was difficult to investigate the detailed fundus because of the dense cataract. **(B)** Optical coherence tomography showed the macular retinal detachment and FVM. **(C)** B-scan ultrasonography detected possible retinal detachment associated with FVM

**Fig. 2:**
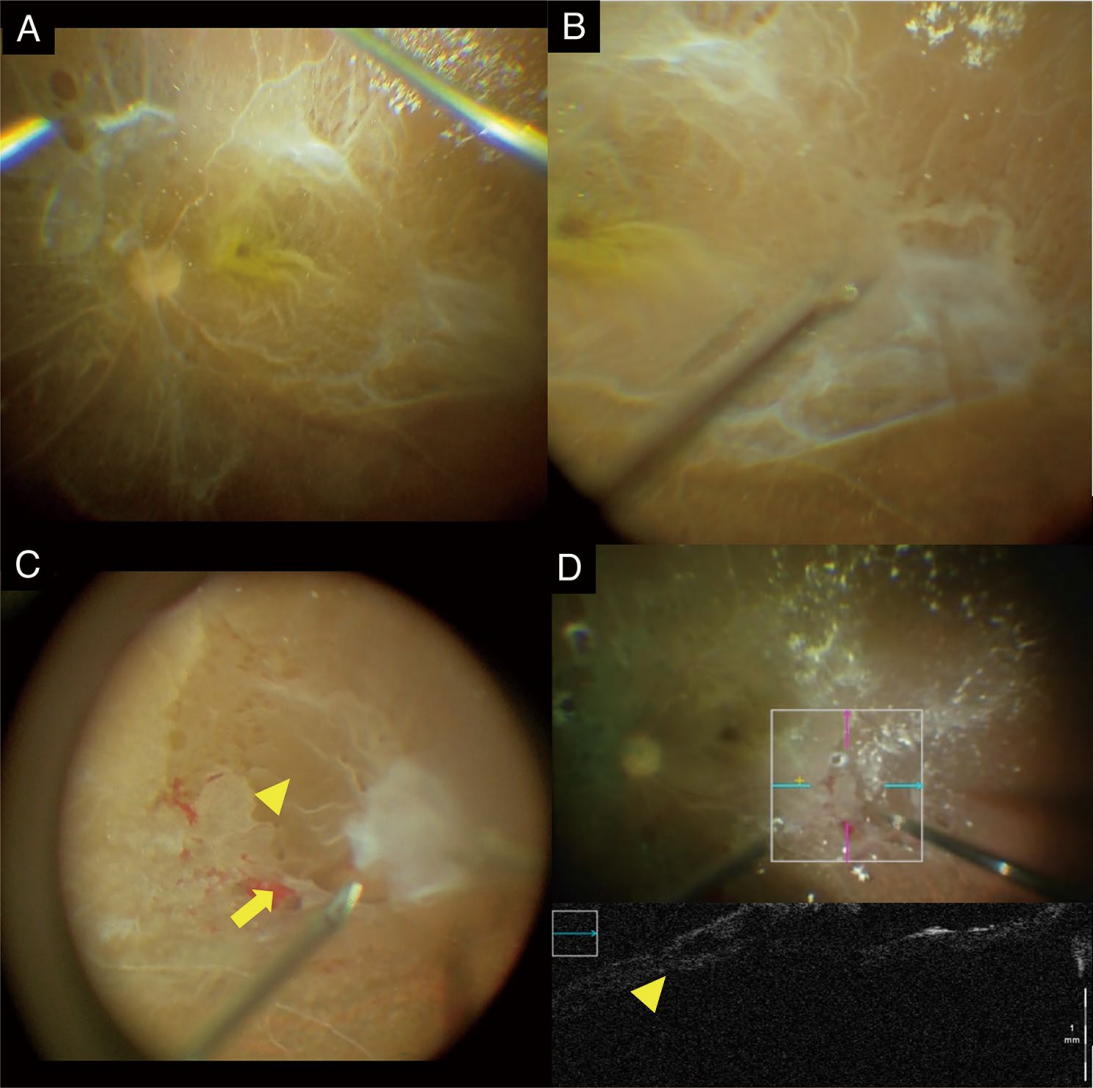
Intraoperative images. **(A)** Multiple holes and severe ischemia were discovered intraoperatively, **(B)** during membrane delamination from the inferior temporal retina. **(C)** The inner retina (arrowhead) was separated from the outer retina (arrow). **(D)** Intraoperative optical coherence tomography showed a space between the inner and outer retina in the detached retina (arrowhead)

A 68-year-old woman presented with decreased vision in the right eye due to PDR with combined retinal detachment. Although it was difficult to perform a detailed fundus observation because of the dense cataract, partial retinal detachment and FVM were identified (Fig. 1A). The contralateral eye was also in a state of severe PDR, with visual acuity at HM, and suspicion of schisis or TRD. However, at the patient’s strong request, surgery was not performed on this eye.　We decided to perform pars plana vitrectomy combined with phacoemulsification and aspiration (PEA) as B-scan ultrasonography and optical coherence tomography detected suspicious large extent of retinal detachment and FVM (Fig. 1B, C). The details of our method are described below and in the supplemental video (supplemental video.mp4). After PEA, pars plana vitrectomy was performed using a 25-gauge vitrectomy system (Constellation Vitrectomy System; Alcon Laboratories, Inc, Fort Worth, TX) and twin light chandelier (DORC, Zuidland, Holand). When core vitrectomy was performed, multiple holes, large retinal detachment extending to all quadrants, and vascular sheathing were found (Fig. 2A). As peripheral posterior vitreous detachment (PVD) had already occurred, the peripheral vitreous was subsequently removed, and delamination of the FVM with TRD was performed from the temporal side (Fig. 2B). During membrane delamination, the space between the outer and inner retina, and not between the retina and FVM, was observed under FVM (Fig. 2C). However, due to the presence of the FVM in the schisis area, there was difficulty separating the inner and outer retina. Therefore, membrane removal was resumed from the attached retina using the bimanual technique. When the FVM was entirely removed, intraoperative optical coherence tomography (iOCT) revealed a space between the inner and outer retina in retinal detachment (Fig. 2D). The inner retina, separated from the outer retina, was removed using a vitreous cutter to prevent potential traction due to postoperative membrane recurrence, as it was disconnected from photoreceptor cells and deemed to not contribute to visual function. After performing fluid-to-gas exchange and photocoagulation, air to 20% sulfur hexafluoride (SF6) gas was exchanged at the end of the surgery.

**Fig. 3:**
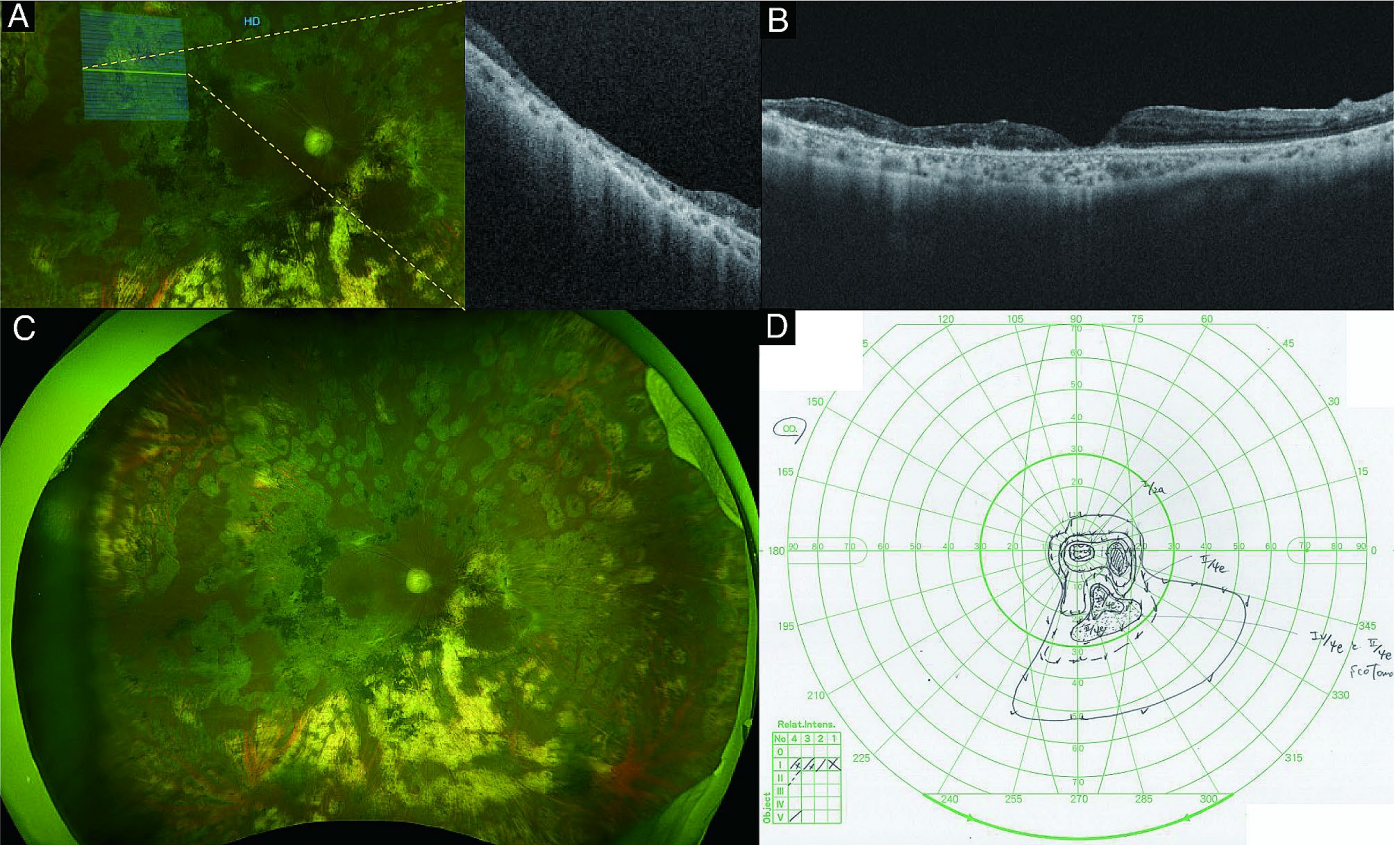
Postoperative images and visual field. **(A)** Postoperatively at 23 months, the swept-source optical coherence tomography (SS-OCT) showed that the retina in which the inner retina was removed became thinner than the other site of retina. **(B-C)** Postoperatively at 26 months, the retinal reattachment was successfully achieved without any complications such as recurrence of retinal detachment in wide-field image and SS-OCT images. **(D)** Postoperatively at 26 months, Goldmann perimetry showed the residual small visual field of the attached retina

Postoperatively at 23 months, a scanning laser ophthalmoscope-Ultra Widefield (UWF) imaging device with integrated full-field OCT (Optos Silverstone; Optos PLC; Dunfermline, UK) showed that the area complicated by retinal separation was thinner than the surrounding area due to removal of the inner retina (Fig. 3A). Finally, retinal reattachment was achieved after this surgery, and the visual acuity improved to 20/40 26 months after surgery (Fig. 3B, C, D).

## Discussion and conclusions

This report is of our intraoperative suspicion of a separation of the inner and outer retina as a cause of tractional retinal laxity, which was confirmed using iOCT. Preoperative OCT often shows tractional retinoschisis in patients with PDR and TRD [4, 6]. Kim et al. reported that diabetic tractional retinal elevation may progress to TRD or tractional retinoschisis [4]. Moreover, a histopathological study showed that retinoschisis is combined with retinal detachment [7]. Specifically, the study investigated four globes with elevated retina with PDR, in which two of four cases were complicated with retinal detachment and retinal elevation, whereas the other two cases were combined only with retinoschisis [7]. In this case, the area of elevated retina was large, and detached retina was possibly associated with retinoschisis. Although we cannot rule out the possibility that the congenital schisis is complicated by FVM, providing definitive evidence for this is difficult. Moreover, given that there was no prior diagnosis of congenital schisis at the previous institution, it is more probable that the schisis is due to FVM rather than being congenital.

Regarding surgery for PDR, a procedure to remove FVM is the most difficult for tractional and rhegmatogenous retinal detachment, because the detached retina is pressed against the FVM. Thus, a vitreoretinal instrument such as scissors or cutter would be unlikely inserted toward the space of retinoschisis during surgery; however, in this case, retinal holes near the arcade were merged into the site of retinoschisis. Moreover, the retinal holes may have been connected to the space of retinoschisis, and the space between the retina and FVM may have been smaller than the space between the retinoschisis. Hence, membrane delamination presumably extended stray into the space between the inner and outer retinal layers through retinoschisis and multiple retinal holes. The patient had sclerosed vessels in all quadrants implying ischemic atrophic retina. Presence of such retina with underlying tractional schisis can be a risk factor for inner-outer retina separation during delamination. It remains to be determined whether segmentation rather than peeling or controlled bimanual peeling in the case of sticky membranes, might be preferable. Additionally, it should be noted that even highly skilled retinal surgeons can encounter issues, one of which may be caused by indiscriminately pulling on the membrane.

To prevent postoperative TRD, we removed the inner retina within the separation between inner and outer retina. In congenital schisis, the inner schisis wall must be removed because the residual posterior cortical vitreous overlying the inner schisis will cause TRD [8]. Given the elevated perceived risk of developing postoperative proliferative membranes in severe PDR compared to other conditions, the inner schisis should be removed, as is done in cases of congenital schisis.

However, as evidenced by our case, unintentional penetration can occur into the space between the inner and outer retina layers. If such intrusion goes unnoticed early on, it could lead to the enlargement of disconnect between the inner and outer layers, resulting in visual deterioration, thus utmost caution is imperative.

Therefore, in cases where tractional retinoschisis due to FVM in PDR is combined with retinal holes, careful membrane delamination is necessary. In such cases, it may be preferable to avoid delamination altogether, opting instead for segmentation, or to perform controlled bimanual delamination when the membranes are densely adherent to the underlying retina. As previously reported, Visual function may benefit from vitreous surgery despite residual macular abnormalities in some cases [9]. Therefore, opting for segmentation alone without forcibly peeling can also be considered a viable option. And the detailed evaluation of space (e.g., between inner and outer retina or between FVM and retina) is crucial during the surgery.

### Electronic supplementary material

Below is the link to the electronic supplementary material.

Supplementary Material 1. Supplemental Video. When core vitrectomy was performed after phacoemulsification and aspiration, multiple holes, large retinal detachment extending to all quadrants, and vascular sheathing were found. Peripheral vitreous was subsequently removed and delamination of the fibrovascular membrane (FVM) with tractional retinal detachment (TRD) was performed from the temporal side. During membrane delamination, the space between the outer and inner retina, and not between the retina and FVM, was observed under FVM. Due to the difficulty separating the inner and outer retina, which was adhered to FVM, membrane removal was resumed from the attached retina using the bimanual technique. When the FVM was entirely removed, the inner retina separated from the outer retina was removed using vitreous cutter to prevent postoperative TRD. After performing fluid-to-gas exchange and photocoagulation, air to 20% sulfur hexafluoride gas was exchanged at the end of the surgery.

## Data Availability

All data supporting our findings are provided within the manuscript.

## Declarations

### Declarations

This study was performed in accordance with the Declaration of Helsinki.

## Abbreviations

PDR: Proliferative diabetic retinopathy
FVM: Fibrovascular membrane
MTRD: Macular tractional retinal detachment
TRD: Tractional retinal detachment
PEA: Phacoemulsification and aspiration
iOCT: Intraoperative optical coherence tomography
SF6: Sulfur hexafluoride
